# Sagittal Spinopelvic Alignment and Mobility Are Not Associated With Low Back Pain in Healthcare Workers

**DOI:** 10.7759/cureus.97778

**Published:** 2025-11-25

**Authors:** Hiroyuki Tokuyasu, Mitsuru Takemoto, Claudio Vergari, Hiroshi Kanoe, Youngwoo Kim

**Affiliations:** 1 Department of Rehabilitation, Kyoto City Hospital, Kyoto, JPN; 2 Department of Orthopaedic Surgery, Kyoto City Hospital, Kyoto, JPN; 3 Department of Biomechanics/Human Movement Analysis, Arts et Métiers Institute of Technology, École Polytechnique Féminine (EPF) Engineering School, Université Sorbonne Paris Nord, Institut de Biomécanique Humaine Georges Charpak (IBHGC), Paris, FRA; 4 Department of Orthopaedics, Kyoto City Hospital, Kyoto, JPN

**Keywords:** healthcare workers, hip pain, low back pain, spinopelvic alignment, spinopelvic mobility

## Abstract

Background

Healthcare workers are frequently exposed to repetitive physical demands and suboptimal postures, which may affect spinopelvic alignment and mobility. However, it remains unclear whether these parameters differ among healthcare occupations and how they relate to low back pain (LBP).

Methodology

This single-center, cross-sectional study included 169 healthcare workers. Spinopelvic alignment and mobility were assessed using functional lateral radiographs in four postures. Patient-reported outcome measures were assessed using the Oswestry Disability Index, the Oxford Hip Score, and the Numerical Rating Scale for LBP and hip pain. Spinopelvic alignment and mobility were analyzed by occupation, and factors related to disability due to LBP were examined.

Results

LBP was present in 38% and hip pain in 11% of participants, with higher LBP prevalence among nurses and nursing assistants compared to other healthcare workers. Significant differences were observed only in pelvic-femoral angle (p < 0.01) and hip angle (p < 0.05) in a relaxed-seated position across occupations, but after adjusting for age, sex, body mass index (BMI), LBP, and hip pain, no significant differences were found in spinopelvic alignment and mobility among occupations. Age and BMI as independent factors were associated with LBP-related disability (p < 0.05 for both).

Conclusions

Healthcare workers in nursing-related professions show a higher prevalence of LBP than those in other healthcare occupations. This LBP was not related to spinopelvic malalignment or mobility restrictions that could be detected through radiographic evaluation. Regular health checkups to identify staff with LBP or hip pain, even in the absence of organic disease, may help establish preventive strategies for reducing LBP and improving health literacy.

## Introduction

Low back pain (LBP) is among the most frequent musculoskeletal conditions, with lifetime prevalence estimated between 60% and 80% [1,2]. LBP is not a disease itself but a clinical symptom that may arise from a wide range of factors [3,4]. From a physiological perspective, its biological contributors include disc degeneration, spinal stenosis, facet joint osteoarthritis, and muscle imbalance [5]. Psychological factors such as stress, anxiety, and depression, as well as social determinants such as occupational demands, socioeconomic status, and interpersonal support, also play a significant role in the onset and persistence of symptoms [6]. These conditions are often multifactorial and interrelated, making the etiology of LBP complex [6].

The prevalence of LBP among healthcare workers is high, ranging from 39% to 80% [7-10], and is an important issue that must be addressed. Previous literature has categorized the potential risk factors for LBP into the following three major groups: (a) individual characteristics, including age and body weight; (b) mechanical exposures, such as heavy lifting, awkward postures, and whole-body vibration; and (c) psychosocial factors, including perceived control and job satisfaction [8]. In addition, spinal alignment and posture (e.g., lumbar lordosis, sagittal vertical axis, and scoliosis) have also been documented as important biomechanical factors associated with LBP [5]. Specifically, repetitive physical activities such as patient handling, transfers, and posture maintenance, often performed with lumbar flexion or trunk rotation in suboptimal ergonomic conditions, are thought to contribute significantly to the development of LBP in these populations [11].

In the field of occupational health, the aging workforce, driven by a declining labor population and delayed retirement, has introduced new challenges. These include decreased physical capacity, increased rates of chronic diseases, and rising mental health concerns, all of which contribute to reduced work productivity. Indeed, LBP is responsible for approximately 149 million lost workdays each year in the United States [12]. The global economic burden of it is estimated at around USD 100 billion annually, with nearly two-thirds of the cost attributed to lost wages and reduced productivity [13]. These findings highlight the negative effects of LBP on the economy and public health. From a workplace safety and health perspective, healthcare institutions need to recognize the economic implications of LBP and implement effective preventive measures.

A recent study has suggested that while the lumbar lordosis angle may remain unchanged in individuals with LBP, their lumbar mobility tends to decrease [14]. Furthermore, given that healthcare workers are required to assume specific postures and perform repetitive movements according to their occupational duties, spinopelvic alignment and mobility may also vary across job types. However, it is unclear whether spinopelvic alignment and mobility differ among healthcare professionals by occupation and are related to LBP.

Therefore, the aim of this study was twofold: (1) to investigate the prevalence of LBP and hip joint pain among healthcare professionals using patient-reported outcome measures (PROMs), and (2) to evaluate spinopelvic alignment and mobility using radiographic assessment by occupations to identify factors associated with LBP in this population.

## Materials and methods

Study design and volunteers

**Figure 1:**
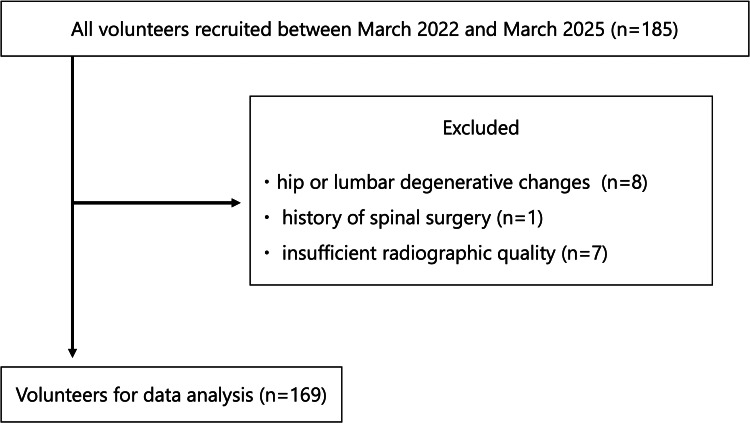
Flowchart of volunteers.

This was a single-center, cross-sectional study conducted between March 2022 and March 2025. Of the 185 volunteers initially enrolled, 16 were excluded according to the following criteria: (1) the presence of hip joint abnormalities, including joint space narrowing or osteophyte formation (Tönnis grade ≥2), as assessed on anteroposterior hip radiographs [15]; (2) significant lumbar pathology, such as advanced disc degeneration (Kellgren-Lawrence grade ≥3) [16], spondylolisthesis (Meyerding grade ≥2) [17], or scoliosis (>30°), as observed on anterior and lateral lumbar radiographs; (3) a history of hip or spinal surgery; and (4) age <20 years. As a result, eight volunteers were excluded because of hip or lumbar degenerative changes, one was excluded due to a history of spinal surgery, and seven were excluded due to insufficient radiographic quality, leaving 169 volunteers for the final analysis (Figure 1).

All participants were healthcare workers employed at the hospital. This study was approved by the institutional review board of our hospital (approval number: 621) and was conducted in compliance with the 2008 Declaration of Helsinki.

Radiographic assessment

**Figure 2:**
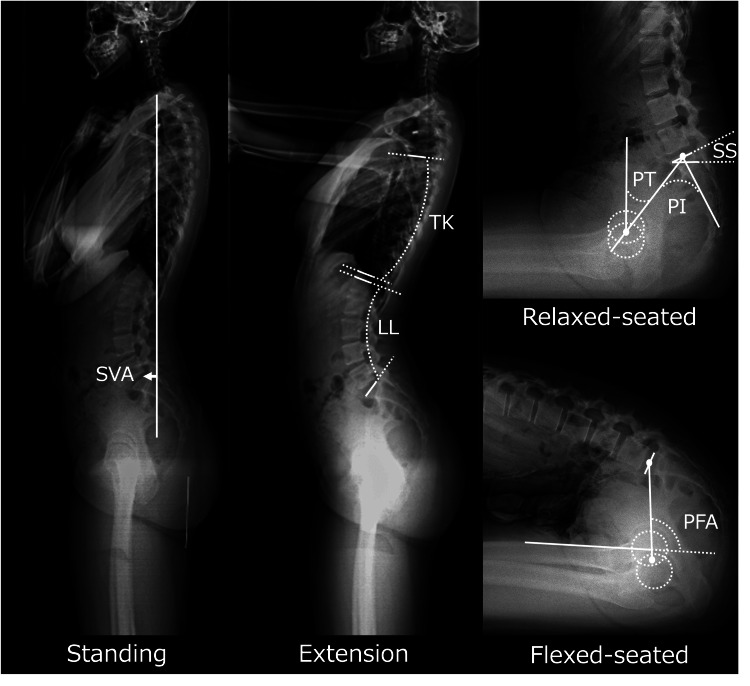
Schematic representation of radiographic analysis for spinopelvic parameters using functional lateral radiographs. The parameters assessed include pelvic incidence (PI), lumbar lordosis from L1 to S1 (LL), sacral slope (SS), pelvic tilt (PT), pelvic-femoral angle (PFA), and the difference between pelvic incidence and lumbar lordosis (PI-LL).

Functional lateral radiographs were obtained in the following four postures: free-standing, standing in extension, relaxed-seated, and flexed-seated positions (Figure 2). For the standing radiograph, patients were instructed to stand upright, gaze forward, and place their fists on their clavicles [18]. For the extension, patients were instructed to grasp a horizontal bar positioned slightly above shoulder level and extend their pelvis and spine to the maximum extent possible [18]. The relaxed-seated position was defined as a posture with the hips and knees flexed at 90°, with both femora parallel to the floor, while seated on a height-adjustable chair. The flexed-seated position was defined as a sitting posture with the femora parallel to the floor and the trunk maximally leaned forward [19-21] (Figure 2). Spinopelvic alignment parameters were measured from these radiographs, including sagittal vertical index (SVA), T4-T12 thoracic kyphosis (TK), pelvic incidence (PI), lumbar lordosis from L1 to S1 (LL), sacral slope (SS), pelvic tilt (PT), pelvic-femoral angle (PFA), and the mismatch between pelvic incidence and lumbar lordosis (PI-LL). TK and LL were measured using the Cobb method, which has been widely adopted in previous studies on spinopelvic alignment [19-21]. PFA was measured as the angle between the proximal femoral axis and the line connecting the center of the femoral heads and the midpoint of the sacral endplate [19-21] (Figure 2).

The hip angle (HipA), adjusted for PI, represents femoral flexion relative to the pelvis and provides a quantitative measure of hip flexion, similar to physical examination in the supine position [20]. Interobserver reliability, as reported in a previous study, was assessed using the intraclass correlation coefficient (ICC), demonstrating excellent agreement of 0.850 to 0.978 [20].

Lumbar mobility was defined as the amount of change in LL between the standing and relaxed-seated or flexed-seated positions (ΔLL). Hip mobility was defined as the amount of change in PFA between the standing and relaxed-seated or flexed-seated positions (ΔPFA).

Patient-reported outcome measures

This study used the Oswestry Disability Index (ODI) and the Oxford Hip Score (OHS) as PROMs. The ODI is one of the most widely used disease-specific rating scales for low back disorders in the world and has already been verified for its validity [22,23]. The ODI consists of 10 questions concerning daily life, each of which is scored on a six-point scale from 0 to 5, with the higher the score, the greater the severity of the disease. Scores are expressed as a percentage, with the total score for each of the 10 items divided by the full score of 50. The total score is then converted to a percentage by dividing by the maximum score of 50. A score of 0%-20% indicates minimal disability; 21%-40% indicates moderate disability; 41%-60% indicates severe disability; 61%-80% indicates crippling back pain; and 81%-100% suggests that the patient may be either bed-bound or exaggerating symptoms. The OHS is a PROM consisting of 12 items that assess pain and physical function related to the hip joint. Each item is rated on a five-point scale from 0 to 4, with a total score ranging from 0 to 48. A higher score indicates better hip function and less pain. The OHS has been validated as a reliable and responsive instrument for evaluating clinical outcomes in hip disorders, and is widely used in clinical settings [24,25]. Both the ODI and the OHS are available for non-commercial academic use without a licensing fee through Mapi Research Trust and Oxford University Innovation, respectively. The presence or absence of LBP and hip pain was assessed using the Numerical Rating Scale (NRS) [26], with a score of ≥3 considered indicative of pain and a score of <3 classified as no pain [27]. The NRS is a freely available, non-proprietary tool widely used in clinical and research settings, and no license is required for its use. The presence of functional disability related to LBP was assessed using the ODI, with a score of >20% indicating the presence of disability.

Statistical analysis

A post hoc power analysis was conducted using G*Power version 3.1.9.7 (Heinrich Heine University, Düsseldorf, Germany), which indicated that the statistical power for the present sample size (n = 169) was 0.73 (1−β = 0.73, α = 0.05). In addition, a post hoc power analysis for the subgroup comparison (independent two-sample t-test, n₁ = 20, n₂ = 149, α = 0.05, two-tailed) demonstrated an achieved power of 0.53, suggesting that the subgroup analysis may have been underpowered to detect small-to-moderate differences. Normality was assessed using the Shapiro-Wilk test. An independent samples t-test or Wilcoxon signed-rank test was performed to compare volunteers with and without LBP-related disability. Categorical data were evaluated using the chi-square test. Comparisons of spinopelvic alignment and mobility among different occupations were conducted using one-way analysis of variance, with Tukey’s post hoc test applied for multiple comparisons when significant differences were observed. Multivariable logistic regression analysis was performed to examine factors affecting LBP-related disability. Statistical significance was set at a p-value <0.05, and data were reported as mean (range). Statistical analysis was performed using JMP Student Edition version 18 (SAS Institute Inc., Cary, NC, USA).

## Results

Study cohort

Cohort demographics are reported in Table 1. The study cohort comprised 111 (66%) women and 58 (34%) men, with a mean age of 37 ± 11 years (range = 21 to 69 years). The mean body mass index (BMI) was 22 ± 3 kg/m² (range = 16 to 40 kg/m²). Groups were formed according to participant occupations, i.e., doctor, nurse, nurse assistant, therapist, and others. A significant difference was observed in age and BMI (p < 0.05 for both) and in sex and LBP (p < 0.01 for both) (Table 1).

**Table 1:** Demographic characteristics of the study participants. Continuous variables are presented as mean ± standard deviation (SD), with ranges shown in parentheses. Categorical variables, including the prevalence of LBP and hip pain, are expressed as numbers and percentages (n, %). Differences among occupational groups were analyzed using one-way analysis of variance with Tukey’s post hoc test for continuous variables, and chi-square tests for categorical variables. No statistical analyses were performed regarding the prevalence of LBP and hip pain. ^a^: Significant difference between nursing assistants and therapists. ^b^: Significant difference between nurses and doctors. ^c^: Significant difference between nursing assistants and doctors. ^d^: Significant difference between others and therapists. ^e^: Significant difference between nurses and therapists. ^f^: Significant difference between others and doctors. *: P < 0.05. **: P < 0.01. LBP = low back pain; ODI = Oswestry Disability Index; OHS = Oxford Hip Score; NRS = Numerical Rating Scale

	Whole (n = 169)	Doctors (n = 30)	Nurses (n = 74)	Nursing assistants (n = 25)	Therapists (n = 13)	Others (n = 27)	Statistical value	P-value
Age (years)	37 ± 11 (21 to 69)	36 ± 11 (24 to 63)	37 ± 10 (23 to 57)	42 ± 12 (21 to 69)	30 ± 7 (23 to 48)	38 ± 9 (25 to 59)	F = 3.176	*^a^
Sex							χ^2^ = 78.107	**
Female (%)	111 (66%)	5 (17%)	66 (89%)	24 (96%)	4 (31%)	12 (44%)		
Male (%)	58 (34%)	25 (83%)	8 (11%)	1 (4%)	9 (69%)	15 (56%)		
BMI (kg/m^2^)	22 ± 3 (16 to 40)	23 ± 2 (19 to 29)	21 ± 3 (17 to 31)	21 ± 5 (16 to 40)	21 ± 2 (18 to 24)	22 ± 2 (18 to 25)	F = 2.709	*^b^
LBP (NRS: 0–10)	2 ± 2 (0 to 9)	1 ± 1 (0 to 5)	2 ± 2 (0 to 8)	3 ± 3 (0 to 7)	0 ± 0 (0 to 2)	3 ± 3 (0 to 9)	F = 6.926	**^a,c,d,e,f^
Hip pain (NRS: 0–10)	1 ± 1 (0 to 9)	0 ± 1 (0 to 3)	1 ± 1 (0 to 6)	1 ± 2 (0 to 7)	0 ± 0 (0 to 0)	1 ± 2 (0 to 9)	F = 1.760	0.139
LBP (%)	64 (38%)	5 (17%)	32 (43%)	16 (64%)	0 (0%)	11 (41%)	-	-
Hip pain (%)	18 (11%)	1 (3%)	9 (12%)	5 (20%)	0 (0%)	3 (11%)	-	-
LBP + hip pain (%)	16 (9%)	0 (0%)	9 (12%)	5 (20%)	0 (0%)	2 (7%)	-	-
ODI (%)	7 ± 8 (0 to 46)	6 ± 10 (0 to 46)	6 ± 8 (0 to 34)	10 ± 8 (0 to 26)	7 ± 10 (0 to 36)	6 ± 8 (0 to 28)	F = 1.440	0.223
OHS (score)	47 ± 2 (34 to 48)	47 ± 3 (34 to 48)	47 ± 2 (38 to 48)	47 ± 2 (39 to 48)	47 ± 2 (41 to 48)	47 ± 1 (43 to 48)	F = 0.706	0.589

Prevalence of low back pain and hip pain by occupation

The overall prevalence of LBP among hospital-based healthcare workers was 38%. When stratified by occupation, the prevalence of LBP was particularly high among nurses (43%) and nursing assistants (63%) (Table 1). The prevalence of hip pain across all participants was 11%, and 9% of the staff reported experiencing both LBP and hip pain simultaneously (Table 1).

Spinopelvic and hip alignment and mobility by occupation

Significant differences among occupational groups were observed only in relaxed-seated PFA and HipA (p < 0.05) (Table 2).

**Table 2:** Spinopelvic alignment and mobility by occupation. The values are described as means and standard deviations, and the ranges are shown in parentheses. Statistical comparisons among occupations were performed using one-way analysis of variance (ANOVA), followed by Tukey’s post hoc test for multiple comparisons (F and P value shown). ^a^: Significant difference between nurses and others. ^b^: Significant difference between nurses and therapists. *: P < 0.05. **: P < 0.01. SVA = sagittal vertical index; TK = T4-T12 thoracic kyphosis; PI = pelvic incidence; LL = L1-S1 lumbar lordosis; SS = sacral slope; PT = pelvic tilt; PFA = pelvic-femoral angle; HipA = hip angle; PI-LL = pelvic incidence minus lumbar lordosis

Parameter	Doctors	Nurses	Nursing assistants	Therapists	Other	F-value	P-value
Standing							
SVA (mm)	3 ± 30 (-31 to 116)	-3 ± 31 (-55 to 120)	-3 ± 32 (-57 to 67)	-12 ± 21 (-44 to 20)	0 ± 25 (-38 to 66)	0.722	0.578
TK (°)	28 ± 9 (10 to 50)	31 ± 9 (9 to 49)	29 ± 8 (11 to 43)	32 ± 13 (5 to 47)	31 ± 8 (17 to 44)	0.800	0.527
LL (°)	53 ± 8 (31 to 63)	53 ± 11 (22 to 75)	54 ± 9 (34 to 73)	56 ± 10 (41 to 77)	51 ± 12 (25 to 74)	0.435	0.783
PI (°)	52 ± 11 (31 to 78)	52 ± 11 (29 to 76)	52 ± 11 (32 to 72)	49 ± 12 (27 to 67)	50 ± 10 (31 to 71)	0.401	0.808
PI-LL (°)	-1 ± 10 (-16 to 26)	-1 ± 10 (-22 to 28)	-2 ± 12 (-25 to 19)	-7 ± 10 (-22 to 9)	-1 ± 7 (-15 to 12)	0.985	0.417
PT (°)	13 ± 8 (0 to 32)	14 ± 7 (-4 to 33)	13 ± 9 (-4 to 28)	10 ± 8 (-2 to 26)	12 ± 5 (2 to 22)	0.929	0.449
SS (°)	39 ± 7 (22 to 49)	38 ± 7 (22 to 52)	39 ± 7 (22 to 53)	40 ± 7 (26 to 50)	37 ± 9 (12 to 55)	0.201	0.937
PFA (°)	-8 ± 7 (-22 to 4)	-9 ± 8 (-27 to 9)	-9 ± 8 (-23 to 6)	-5 ± 8 (-21 to 4)	-7 ± 7 (-23 to 9)	1.173	0.325
HipA (°)	5 ± 6 (-8 to 14)	3 ± 6 (-11 to 17)	3 ± 6 (-9 to 12)	6 ± 5 (-4 to 12)	4 ± 7 (-3 to 16)	1.087	0.365
Extension							
TK (°)	24 ± 12 (-4 to 52)	28 ± 12 (-2 to 54)	29 ± 11 (2 to 53)	29 ± 10 (9 to 42)	27 ± 13 (0 to 47)	0.826	0.511
LL (°)	61 ± 9 (42 to 75)	61 ± 10 (35 to 88)	62 ± 9 (45 to 82)	66 ± 9 (49 to 81)	61 ± 14 (35 to 86)	0.595	0.667
PT (°)	15 ± 10 (-1 to 39)	16 ± 8 (-2 to 35)	16 ± 9 (0 to 34)	10 ± 9 (-5 to 28)	15 ± 8 (0 to 27)	1.805	0.130
SS (°)	37 ± 10 (17 to 58)	35 ± 8 (16 to 54)	35 ± 7 (25 to 48)	38 ± 8 (24 to 49)	35 ± 10 (10 to 58)	0.661	0.620
PFA (°)	-12 ± 10 (-29 to 6)	-14 ± 8 (-34 to 6)	-15 ± 9 (-31 to 0)	-8 ± 10 (-29 to 11)	-12 ± 8 (-25 to 10)	1.617	0.172
HipA (°)	0 ± 9 (-17 to 21)	-2 ± 7 (-16 to 16)	-3 ± 7 (-14 to 12)	3 ± 7 (-12 to 14)	-1 ± 8 (-14 to 17)	1.554	0.189
Relaxed-seated							
LL (°)	33 ± 12 (15 to 63)	32 ± 15 (1 to 60)	37 ± 11 (13 to 61)	36 ± 13 (16 to 57)	36 ± 12 (9 to 55)	1.051	0.383
PT (°)	25 ± 10 (6 to 44)	26 ± 11 (-3 to 55)	21 ± 11 (1 to 38)	19 ± 8 (5 to 31)	21 ± 9 (0 to 38)	2.286	0.062
SS (°)	28 ± 9 (12 to 50)	28 ± 10 (0 to 52)	30 ± 8 (10 to 51)	30 ± 10 (9 to 49)	30 ± 11 (4 to 55)	0.548	0.701
PFA (°)	70 ± 10 (54 to 88)	67 ± 13 (32 to 99)	70 ± 12 (54 to 89)	76 ± 10 (59 to 95)	74 ± 10 (51 to 92)	3.467	**^a,b^
HipA (°)	82 ± 7 (68 to 96)	79 ± 12 (38 to 103)	82 ± 10 (67 to 100)	88 ± 10 (69 to 104)	86 ± 9 (70 to 102)	3.312	*^a^
Flexed-seated							
LL (°)	-10 ± 10 (-25 to 15)	-9 ± 10 (-29 to 28)	-6 ± 10 (-26 to 13)	-12 ± 11 (-33 to 2)	-10 ± 14 (-32 to 31)	0.876	0.480
PT (°)	-5 ± 11 (-25 to 19)	-9 ± 12 (-38 to 27)	-12 ± 10 (-29 to 13)	-14 ± 10 (-27 to 8)	-9 ± 8 (-30 to 3)	2.353	0.056
SS (°)	57 ± 13 (36 to 85)	63 ± 10 (41 to 91)	64 ± 9 (40 to 80)	63 ± 12 (32 to 82)	59 ± 11 (32 to 83)	2.085	0.085
PFA (°)	98 ± 11 (73 to 120)	101 ± 13 (67 to 124)	102 ± 12 (75 to 124)	109 ± 10 (85 to 122)	104 ± 9 (86 to 124)	2.325	0.059
HipA (°)	111 ± 11 (84 to 137)	113 ± 11 (88 to 136)	115 ± 10 (90 to 133)	121 ± 8 (103 to 134)	115 ± 9 (101 to 135)	2.258	0.065
ΔLL (°)							
Standing/Flexed-seated	62 ± 9 (37 to 81)	61 ± 9 (37 to 79)	60 ± 13 (38 to 81)	68 ± 8 (54 to 79)	61 ± 12 (29 to 81)	1.503	0.204
Extension/Flexed-seated	71 ± 11 (52 to 91)	70 ± 10 (42 to 90)	68 ± 15 (41 to 97)	78 ± 8 (65 to 86)	71 ± 14 (34 to 96)	1.782	0.135
ΔPFA (°)							
Standing/Flexed-seated	106 ± 13 (75 to 136)	110 ± 10 (84 to 132)	111 ± 10 (90 to 133)	115 ± 7 (103 to 129)	111 ± 6 (102 to 126)	2.195	0.072
Extension/Flexed-seated	111 ± 15 (77 to 136)	115 ± 12 (90 to 137)	117 ± 10 (90 to 131)	117 ± 10 (99 to 137)	116 ± 8 (100 to 133)	1.465	0.215
Hip user index [%]							
Standing/Flexed-seated	63 ± 6 (48 to 74)	64 ± 5 (54 to 76)	65 ± 5 (56 to 76)	63 ± 4 (57 to 68)	65 ± 5 (57 to 80)	1.175	0.324
Extension/Flexed-seated	61 ± 6 (46 to 72)	62 ± 4 (51 to 77)	64 ± 5 (53 to 76)	60 ± 3 (54 to 65)	62 ± 6 (51 to 78)	1.521	0.198

However, after adjusting for age, sex, BMI, LBP, and hip pain, no significant differences in relaxed-seated HipA were observed among occupations (p > 0.05) (Table 3). In other words, spinopelvic and hip alignment and mobility do not significantly differ by occupation.

**Table 3:** Multivariable linear regression analysis for factors associated with HipA in the relaxed-seated position. Multivariable linear regression analysis was performed to identify independent factors associated with HipA in the relaxed-seated position. Values are presented as regression coefficients (β) with SE, 95% CI, and p-value. Male was used as the reference group for sex, and nurse was used as the reference group for occupation. *: P < 0.05. **: P < 0.01. BMI = body mass index; LBP = low back pain; HipA = hip angle; SE = standard error; CI = confidence interval

Variable	β	SE	95%CI	P value
Age	0.07	0.97	-0.07 to 0.22	0.335
Female	-3.22	1.06	-5.31 to -1.13	**
BMI	-1.21	0.25	-1.71 to -0.72	**
Occupation: Doctor	-1.47	1.87	-5.17 to 2.22	0.432
Occupation: Nursing assistant	0.75	2.03	-3.25 to 4.76	0.711
Occupation: Therapist	2.26	2.41	-2.50 to 7.02	0.350
Occupation: Other	1.17	1.71	-2.23 to 4.57	0.497
LBP	0.18	0.44	-0.68 to 1.04	0.686
Hip pain	-0.42	0.60	-1.59 to 0.76	0.488

Univariable and multivariable analyses of factors associated with disability due to low back pain

The univariable analysis revealed that age was the only significant difference between the two groups with and without disability due to LBP (p < 0.05) (Table 4).

**Table 4:** Spinopelvic alignment and mobility according to with or without disability related to low back pain. The values are described as means and standard deviations, and the ranges are shown in parentheses. Statistical analysis was performed as follows: ^a^, independent-samples t-test; ^b^, Wilcoxon rank-sum test; ^c^, chi-square test. The corresponding test statistics (t, Z, or χ² values) are presented in the table along with p-values. *: P < 0.05. **: P < 0.01. BMI = body mass index; SVA = sagittal vertical index; TK = T4-T12 thoracic kyphosis; PI = pelvic incidence; LL = L1-S1 lumbar lordosis; SS = sacral slope; PT = pelvic tilt; PFA = pelvic-femoral angle; HipA = hip angle; PI-LL = pelvic incidence minus lumbar lordosis

Parameter	With disability (n = 20)	Without disability (n = 149)	Statistical value	P-value
Age (years)	42 ± 9 (25 to 57)	36 ± 11 (21 to 69)	t = 2.479	*^b^
Sex			χ^2^ = 0.915	0.339^c^
Female, n (%)	15 (64%)	96 (75%)		
Male, n (%)	5 (36%)	53 (25%)		
BMI (kg/m^2^)	23 ± 5 (18 to 40)	22 ± 3 (16 to 31)	Z = 1.467	0.142^b^
Occupation			χ^2^ = 1.996	0.737^c^
Doctor, n (%)	3 (15%)	27 (18%)		
Nurse, n (%)	8 (40%)	66 (44%)		
Nurse assistant, n (%)	5 (25%)	20 (14%)		
Therapist, n (%)	1 (5%)	12 (8%)		
Other, n (%)	3 (15%)	24 (16%)		
Standing				
SVA (mm)	-4 ± 24 (-44 to 39)	-2 ± 30 (-57 to 120)	Z = 0.085	0.932^b^
TK (°)	31 ± 9 (11 to 49)	30 ± 9 (5 to 50)	t = 0.267	0.790^a^
LL (°)	55 ± 11 (32 to 73)	53 ± 10 (22 to 77)	t = 1.067	0.288^a^
PI (°)	53 ± 11 (34 to 76)	51 ± 11 (27 to 78)	t = 0.658	0.512^a^
PI-LL (°)	-2 ± 11 (-20 to 12)	-1 ± 10 (-25 to 28)	t = -0.382	0.703^a^
PT (°)	13 ± 8 (-2 to 24)	13 ± 7 (-4 to 33)	t = -0.408	0.684^a^
SS (°)	41 ± 7 (27 to 52)	38 ± 8 (12 to 55)	Z = 1.252	0.211^b^
PFA (°)	-8 ± 9 (-21 to 9)	-9 ± 7 (-27 to 9)	t = 0.554	0.580^a^
HipA (°)	5 ± 7 (-7 to 17)	3 ± 6 (-13 to 17)	t = 1.126	0.262^a^
Extension				
TK (°)	26 ± 13 (2 to 54)	28 ± 12 (-4 to 53)	t = -0.571	0.569^a^
LL (°)	61 ± 11 (35 to 78)	62 ± 11 (35 to 88)	t = 0.196	0.845^a^
PT (°)	15 ± 10 (-3 to 29)	15 ± 9 (-5 to 39)	t = -0.204	0.839^a^
SS (°)	37 ± 6 (26 to 50)	35 ± 9 (10 to 58)	t = 0.688	0.492^a^
PFA (°)	-13 ± 10 (-30 to 10)	-13 ± 9 (-34 to 11)	t = -0.037	0.970^a^
HipA (°)	-1 ± 8 (-14 to 17)	-1 ± 8 (-17 to 21)	t = 0.293	0.770^a^
Relaxed-seated				
LL (°)	35 ± 10 (17 to 50)	34 ± 14 (1 to 63)	Z = -0.088	0.930^b^
PT (°)	25 ± 11 (5 to 49)	24 ± 11 (-3 to 55)	t = 0.386	0.700^a^
SS (°)	29 ± 7 (20 to 44)	28 ± 10 (0 to 55)	t = 0.271	0.787^a^
PFA (°)	68 ± 13 (41 to 88)	70 ± 12 (32 to 99)	t = -0.644	0.521^a^
HipA (°)	81 ± 10 (62 to 95)	82 ± 11 (38 to 104)	Z = -0.363	0.712^b^
Flexed-seated				
LL (°)	-7 ± 11 (-29 to 11)	-9 ± 11 (-33 to 31)	Z = 1.118	0.264^b^
PT (°)	-8 ± 11 (-25 to 13)	-9 ± 11 (-38 to 27)	t = 0.526	0.599^a^
SS (°)	61 ± 11 (40 to 81)	61 ± 11 (32 to 91)	t = -0.260	0.796^a^
PFA (°)	100 ± 12 (75 to 123)	102 ± 12 (67 to 124)	t = -0.895	0.372^a^
HipA (°)	112 ± 10 (90 to 131)	114 ± 11 (84 to 137)	t = -0.748	0.455^a^
ΔLL (°)				
Standing/Flexed-seated	63 ± 12 (38 to 80)	62 ± 10 (29 to 81)	t = 0.308	0.759^a^
Extension/Flexed-seated	69 ± 11 (48 to 85)	71 ± 12 (34 to 97)	t = -0.492	0.623^a^
ΔPFA (°)				
Standing/Flexed-seated	107 ± 7 (90 to 118)	111 ± 10 (75 to 136)	t = -1.482	0.140^a^
Extension/Flexed-seated	113 ± 10 (90 to 133)	115 ± 12 (77 to 137)	t = -0.880	0.380^a^
Hip user index (%)				
Standing/Flexed-seated	63 ± 5 (57 to 75)	64 ± 5 (48 to 80)	t = -0.730	0.467^a^
Extension/Flexed-seated	62 ± 4 (56 to 71)	62 ± 5 (46 to 78)	t = 0.015	0.988^a^

The multivariable logistic regression analysis revealed that both age and BMI were independently associated with disability due to LBP (p < 0.05 (Table 5).

**Table 5:** Risk factors associated with disability due to low back pain. Multivariable logistic regression analysis was performed to identify factors associated with disability due to low back pain. Lumbar mobility (st-flex): change in lumbar lordosis angle from standing to flexed-seated position. *: P < 0.05. **: P < 0.01. OR = odds ratio; CI = confidence interval; BMI = body mass index

Variable	OR	95% CI	P-value
Age	1.06	1.01–1.12	*
Female	1.97	0.68–6.68	0.22
BMI	1.20	1.04–1.39	*
Lumbar mobility (st-flex)	1.04	0.98–1.10	0.16

## Discussion

This study demonstrated that LBP was prevalent among healthcare workers, with 38% reporting LBP, 11% reporting hip pain, and 9% experiencing pain in both the lumbar spine and hip. Spinopelvic alignment and mobility did not significantly differ across occupational groups, but several factors were associated with disability due to LBP. Healthcare workers are required to maintain specific postures and perform repetitive movements as part of their duties. Consequently, spinopelvic alignment and mobility may differ by occupation, although this has not been well elucidated. This study is the first to assess the prevalence of LBP and hip pain among healthcare workers using PROMs, analyze spinopelvic alignment and mobility across occupational groups, and identify risk factors associated with disability due to LBP. The prevalence of LBP among healthcare workers has been reported in numerous reports at 39%-80% [7-10]. Although the definition of LBP varies among previous studies, making direct comparisons difficult, the prevalence of LBP in this study was 38%. Previous studies have reported that, when examining the prevalence of LBP by occupation, nurses, in particular, have a high prevalence of LBP [28]. Similarly, in this study, the prevalence of LBP was significantly higher in nursing-related professions, such as nurses and nursing assistants, than in other occupational groups. Factors contributing to the high prevalence of LBP among nurses include the repeated performance of tasks such as patient repositioning and transfer assistance, which involve bending and rotating the lumbar spine in poor posture, thereby subjecting the lower back to sustained or repetitive stress [10]. These repetitive physical activities and poor posture may cause variations in spinopelvic alignment and mobility, which could differ depending on the occupation of healthcare workers. However, the results of this study demonstrated that spinopelvic alignment, as well as mobility, do not differ across occupations. This can be attributed to the fact that relatively young and healthy healthcare workers, who are capable of performing their duties, typically present with mild LBP. In these individuals, the influence of radiographically detectable organic disorders, spinopelvic malalignment, or mobility restrictions is minimal. It is also possible that the absence of significant differences in spinopelvic parameters among occupational groups was due to the relatively small sample size or limited variability in the study population. Furthermore, multivariate analysis revealed that age and BMI were factors contributing to disability due to LBP. These findings underscore the importance of addressing both physical and organizational factors in preventing LBP among healthcare workers, particularly nurses. To prevent the onset of LBP among individuals involved in nursing care, it is essential to implement a multifaceted approach. This includes education on proper body mechanics to reduce high physical loads, such as frequent bending, lifting, and patient transfers, as well as workplace modifications aimed at alleviating excessive workloads and psychological stress. Additionally, health promotion programs that encourage weight management and physical activity may be beneficial in mitigating LBP in this population.

The results of this study showed that the prevalence of hip pain was 11%, and 9% of healthcare workers experienced pain in both the lumbar spine and hip joint. Although this prevalence rate was lower than that for LBP, a certain number of cases were identified. These findings highlight that, in addition to LBP, hip pain and concurrent lumbar-hip pain are relevant musculoskeletal issues among healthcare workers. There are numerous reports on the prevalence of LBP among healthcare workers [7-10], but few reports on the prevalence of hip pain or on workers who experience pain in both the lumbar spine and hip. However, considering the current situation where the aging of the workforce is accelerating, the number of workers with hip pain or pain in both the lumbar spine and hip is likely to increase in the future. This may be due to staff members perceiving LBP as hip pain or experiencing hip symptoms due to overwork. Since Macnab et al. proposed the hip-spine syndrome in 1983 [29], numerous studies have shown that the lumbar spine, pelvis, and hip joint are adjacent joints that closely influence each other and affect each other’s pathologies. Indeed, lumbar zygapophyseal joint pain, which is a major contributor to non-specific LBP, has been associated with referred pain in the lateral thigh through the medial branches of the dorsal rami from L3 to S1 and in the groin region through the medial branch of the dorsal ramus at L4 [30]. Consequently, it is possible that some healthcare workers perceived LBP as hip pain. Additionally, healthcare workers frequently perform tasks such as patient repositioning, transfer assistance, and shoe/sock removal assistance, which involve bending forward or squatting, potentially triggering not only LBP but also hip joint pain. Therefore, to improve the working environment in hospitals, it is considered important to identify staff members with LBP or hip pain through regular health checkups, and to provide them with specific improvement methods, even in cases where no organic diseases are present, to establish preventive measures and enhance health literacy.

This study has several limitations. First, it was a single-center study, which may limit the generalizability of our findings to other settings with different patient populations or working environments. Future multicenter studies are needed to confirm these results. Second, the sample included a relatively high proportion of nurses and nursing assistants, with fewer participants from other healthcare professions, such as therapists. Third, the study population consisted mainly of relatively young and healthy female healthcare workers, which may limit the generalizability of our findings to other age groups or occupational populations. Fourth, the evaluation was based solely on radiographs, without the use of MRI or CT, which could identify the underlying causes of LBP or hip pain. Fifth, this study did not assess occupational factors such as work-related postures, frequency of load handling, ergonomic conditions, duration of work, years of experience, time on the job, or psychosocial components, which may also contribute to LBP. Additionally, static radiographic assessments may not fully reflect the dynamic movements involved in real-world activities. Finally, most participants had mild LBP and a low BMI. These methodological improvements and controls would enhance the accuracy and generalizability of future findings on LBP and hip pain among healthcare workers.

## Conclusions

Healthcare workers in nursing-related professions show a higher prevalence of LBP than those in other healthcare occupations. This LBP was not related to spinopelvic malalignment or mobility restrictions that could be detected through radiographic evaluation. Given the aging workforce, it is essential to implement early and periodic screening to identify individuals with LBP and hip pain through routine health checkups and provide targeted interventions, even in the absence of organic disease. Such efforts may help establish preventive measures and improve health literacy among healthcare workers.
